# Advancements in Flexible Nanogenerators: Polyvinylidene Fluoride-Based Nanofiber Utilizing Electrospinning

**DOI:** 10.3390/molecules29153576

**Published:** 2024-07-29

**Authors:** Jin-Uk Yoo, Dong-Hyun Kim, Tae-Min Choi, Eun-Su Jung, Hwa-Rim Lee, Chae-Yeon Lee, Sung-Gyu Pyo

**Affiliations:** School of Integrative Engineering, Chung-Ang University, 84, Heukseok-ro, Dongjak-gu, Seoul 06974, Republic of Korea; wlsdnr5771@naver.com (J.-U.Y.); donghyun0927@naver.com (D.-H.K.); c79411@gmail.com (T.-M.C.); eunsuj@cau.ac.kr (E.-S.J.); ghkfla0725@naver.com (H.-R.L.); chyth1194@naver.com (C.-Y.L.)

**Keywords:** electrospinning, energy harvesting, flexible nanogenerator, PVDF

## Abstract

With the gradual miniaturization of electronic devices and the increasing interest in wearable devices, flexible microelectronics is being actively studied. Owing to the limitations of existing battery systems corresponding to miniaturization, there is a need for flexible alternative power sources. Accordingly, energy harvesting from surrounding environmental systems using fluorinated polymers with piezoelectric properties has received significant attention. Among them, polyvinylidene fluoride (PVDF) and PVDF co-polymers have been researched as representative organo-piezoelectric materials because of their excellent piezoelectric properties, mechanical flexibility, thermal stability, and light weight. Electrospinning is an effective method for fabricating nanofibrous meshes with superior surface-to-volume ratios from polymer solutions. During electrospinning, the polymer solution is subjected to mechanical stretching and in situ poling, corresponding to an external strong electric field. Consequently, the fraction of the piezoelectric β-phase in PVDF can be improved by the electrospinning process, and enhanced harvesting output can be realized. An overview of electrospun piezoelectric fibrous meshes composed of PVDF or PVDF co-polymers to be utilized is presented, and the recent progress in enhancement methods for harvesting output, such as fiber alignment, doping with various nanofillers, and coaxial fibers, is discussed. Additionally, other applications of these meshes as sensors are reviewed.

## 1. Introduction

In the modern civilization pursuing a more convenient life, artificial intelligence and internet of things (IoT) technologies are being evaluated as critical industrial technologies, along with recent developments in 5G communication technology. In addition, advancements in semiconductor manufacturing technology have led to the miniaturization of electronic products, and wearable electronics are gradually gaining attention within this trend. Wearable devices that combine various IoT sensors can improve human health by monitoring vital signs in real time when attached to human skin or clothes. However, the existing electrochemical inorganic battery systems not only make it difficult to secure flexibility characteristics but also cause environmental pollution problems due to the use of heavy metals such as nickel and cobalt and toxic electrolytes, as well as their limited lifespan, safety, and sustainability issues due to miniaturization. Therefore, there is a need for self-powering alternative energy supply systems that are compact, sustainable, lightweight, and flexible [1,2,3].

**Figure 1:**
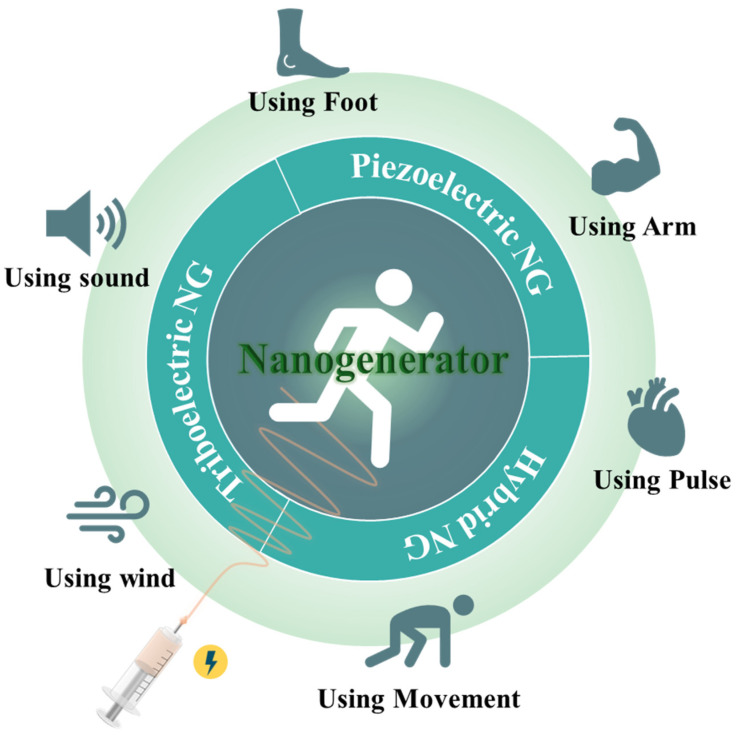
Illustration of the kind of nanogenerators (NGs) such as Piezoelectric NGs, Triboelectric NGs and Hybrid NGs that can produce energy using a variety of methods.

Energy-harvesting technology converts various energy sources, such as solar [4], wind [5], thermal energy [6], and mechanical vibration [7], which are wasted from the environment, into electrical energy that can be used by devices. If such an energy harvester can generate sufficient power to drive a device, it will be possible to build an energy storage system that does not require charging or replacement. Flexible nanogenerator (NG) technology is attracting attention as an alternative energy source that meets the requirements of wearable electronics (Figure 1). Piezoelectric nanogenerators (PENGs) and triboelectric nanogenerators (TENGs) use various mechanical motions of the body to generate electricity. PENGs and TENGs have the advantages of not being restricted by location, weather conditions, or time of day, simple structure, easy fabrication, and a wide selection of materials [8]. Therefore, PENGs and TENGs have been introduced as suitable energy sources for wearable devices, and various studies are being conducted to improve their characteristics and performance [9,10,11,12,13].

**Figure 2:**
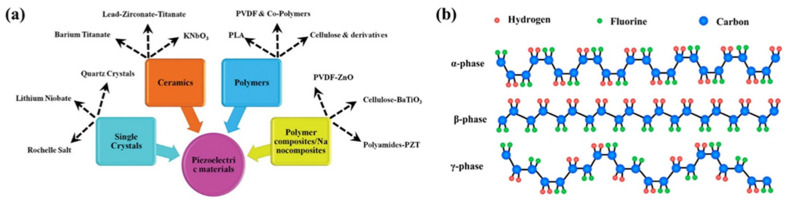
(**a**) Different classes of piezoelectric materials; Reproduced with permission from ref. [14]; Copyright 2019, John Wiley & Sons (**b**) Schematic of chain formation for α-, β-, and γ-phases of polyvinylidene fluoride (PVDF); Reproduced with permission from ref. [17]; Copyright 2018, MDPI.

The piezoelectric effect is a phenomenon in which a potential is generated by the deformation of the crystal structure of a material due to external mechanical stress. Depending on the direction of the stress, the center of mass of the positive and negative ions changes, inducing net polarization, which generates a potential difference at both ends, called the piezoelectric potential. Piezoelectric materials are generally classified as inorganic or polymeric. Representative inorganic piezoelectric materials include single crystals with non-centrosymmetric crystal structures, such as quartz or wurtzite zinc oxide (ZnO), lead zirconate titanate (Pb[Zr_x_Ti_1−x_]O_3_, PZT), barium titanate (BaTiO_3_, BT), and potassium sodium niobate (K_1−x_Na_x_NbNO_3_, KNN), and polycrystalline ceramics consisting of a large number of tiny crystals with random orientations (Figure 2a) [14]. In particular, piezoelectric ceramics are the most actively studied energy harvesters because of their high piezoelectric coefficients, good mechanical properties, and stability. However, lead (Pb) is toxic to humans, harmful to the environment, and crucially brittle, limiting its use in flexible electronics [15,16]. Nonetheless, although organic polymers have a lower piezoelectric coefficient than inorganic materials, they are lightweight, inexpensive, easy to process, biocompatible, and most importantly, flexible [17]. For polymers with piezoelectric characteristics, inherent molecular dipoles must exist within the molecular structure, and these dipoles must be reoriented to a preferred orientation state (called the poling process) by a strong external electric field. The stronger electronegativity of fluorine compared to carbon and hydrogen leads to the formation of inherent dipoles between atoms, making fluoropolymers suitable for use as piezoelectric polymers [18,19,20]. It is a common misconception that the piezoelectric effect and the flexoelectric effect are one and the same. This is because they are both electrical effects produced by the deformation of a material by an externally applied electric field. However, there are subtle differences between these two effects. Flexo-electricity is the property of a dielectric material to produce electrical polarization by stresses caused by strain gradients or electric field gradients [21]. The flexoelectric effect is not limited to polymers; it also includes materials such as Si [22]. Therefore, it is important to be clear and understand the difference with piezoelectricity.

Among the piezoelectric polymers, polyvinylidene fluoride (PVDF) has been actively studied in the field of energy harvesting because of its high piezoelectric coefficient, superior stability, and good mechanical flexibility. Due to the difference in the Van der Waals radius of fluorine (1.35 Å) and the hydrogen atom (1.20 Å), PVDF is a semi-crystalline polymer with five different crystalline phases depending on the orientation of the -CF_2_ and -CH_2_ groups (Figure 2b). Among them, the electroactive β-phase of the all-trans (TT) planar zigzag structure has been utilized in the field of energy generation because it has outstanding electrical characteristics, including piezo-, pyro-, and ferroelectric properties due to the closer packing of polymer chains [23,24]. In detail, the piezoelectricity of PVDF comes from its non-centrosymmetric structure caused by the -CF_3_ and -CH_2_ groups. The non-centrosymmetric structure of PVDF results in an uneven distribution of positive and negative charges and a change in the dipole moment inside the crystal. When external stresses are applied to PVDF, the direction and intensity of polarization of the molecules changes. Therefore, the piezoelectricity changes according to the difference in electronegativity of the functional groups [25,26,27]. In particular, co-polymerization is used as an effective means of inducing the phase transition of PVDF, where the comonomer type and composition ratio determine the crystalline structure and crystallinity of the PVDF co-polymer. Generally, by introducing bulky comonomers into PVDF chains, β-phase formation is promoted by steric hindrance [28]. In addition, nanostructures have been studied to produce better voltage outputs based on these materials. Piezoelectric materials with nanostructures can withstand higher strains than typical materials, allowing for higher voltages. They also exhibit higher energy-conversion efficiencies than bulk materials owing to their high surface-to-volume ratios. Therefore, researchers are actively working toward the utilization of nanoscale piezoelectric materials [29,30,31,32].

**Figure 3:**
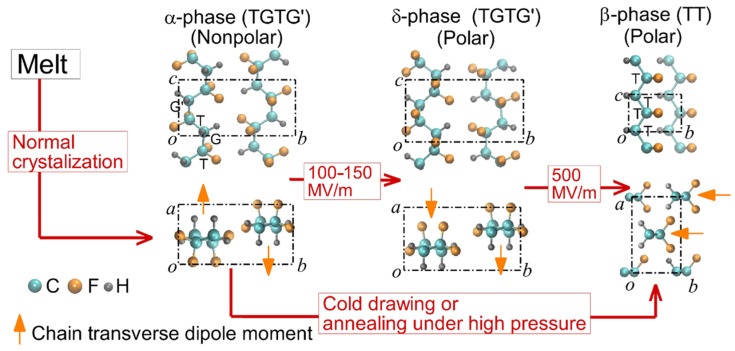
Electric field-induced phase transitions of PVDF; Reproduced with permission from ref. [37]; Copyright 2016, AIP Publishing.

Electrospinning is a simple and efficient technique for the production of continuous nanofibers from polymer solutions. Electrospinning utilizes electrostatic forces to induce spinning in a solution. When a high voltage is applied to the solution, a surface charge is generated on the droplets through the spinneret, and the droplets collapse under conditions above the critical voltage to form a jet. When a strong external electric field overcomes the surface tension, a charged jet is ejected from the tip of the Taylor cone [33,34]. The charged jet undergoes bending (or whipping) instability due to the coupling of the liquid strands by the external electric field and finally exhibits solvent evaporation, inducing the formation of a precipitated continuous thin nanofiber [35]. The final fiber structure has the advantage of being easy to tune for suitable applications because it can be adjusted according to the characteristics of the polymer itself, such as the molecular weight and solubility; the characteristics of the solution, such as viscoelasticity, surface tension, and conductivity, which are changed by the type or concentration of the solvent; and the surrounding environmental characteristics, such as the magnitude of the external electric field, feed rate, electrode geometry, and environmental humidity [36]. Nanofiber meshes can be used in various fields because they exhibit superior properties that are different from those of conventional bulk materials, owing to their large surface area and structural characteristics. For example, electrospinning is an effective way to produce nanoscale PVDF easily and quickly. In particular, PVDF nanofibers produced by electrospinning do not require the post-poling process that is required for conventional piezoelectric materials. Because the PVDF polymer jet was placed in a high electrostatic field during the electrospinning process, the PVDF nanofibers were naturally mechanically stretched and underwent in situ electric poling. As shown in Figure 3, PVDF usually exhibits the morphology of the non-polar α-phase in general, so in order to obtain PVDF in the piezoelectric β-phase, an electrical poling and mechanical stretching process is required to rearrange the dipoles in the crystalline PVDF structure [37]. However, electrospun PVDF mesh has the advantage that the formation of the β-phase can be induced naturally during the formation of nanofibers [38,39,40]. Therefore, the PVDF nanofiber matrix prepared via electrospinning has excellent piezoelectric characteristics, good mechanical flexibility, and stability, making it one of the most suitable materials for use in flexible nanogenerators in wearable electronics. Various methods are available for producing enhanced nanofiber meshes using electrospinning. For example, aligning fibers using a drum-rotating collector [41], producing composite nanofibers by adding additive doping materials to the polymer solution [42], and synthesizing coaxial nanofibers using a coaxial nozzle [43] have been actively studied. The resulting PVDF-based nanofiber matrix can be used not only as a nanogenerator to convert mechanical energy into electrical energy, such as in acoustic energy harvesting [44], but also as a sensor to detect input signals by measuring the harvesting output in reverse, such as in respiratory sensors [45] and human motion sensors [46].

In this study, various experimental variables involved in the fabrication of electrospun PVDF or PVDF co-polymer nanofibers for use as nanogenerators were explored, and the crystallinity and crystalline phase characteristics of the fabricated meshes were examined using various analytical methods, such as FT-IR, X-ray diffraction (XRD), and differential scanning calorimetry (DSC). In addition, various studies on PENGs, TENGs, and hybrid NGs utilizing PVDF nanofiber meshes have been conducted, and various methods to enhance the piezoelectric output have been explored. Finally, examples of applications of PVDF-based nanogenerators as real devices are presented, and a discussion of the prospects for future wearable electronics is provided.

## 2. Fabrication of PVDF-Based Nanofibrous Mesh by Electrospinning

**Figure 4:**
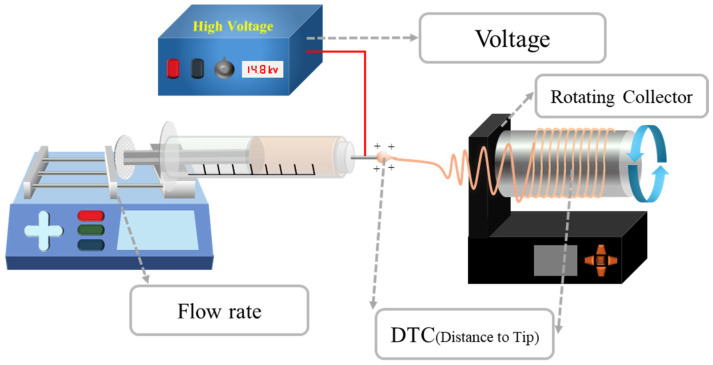
Schematic of electrospinning process and each part name.

As shown in Figure 4, the electrospinning process can produce nanofibers with different properties depending on the experimental conditions. Generally, material properties such as molecular weight, molecular weight distribution, solubility, and solvent vapor pressure, along with process parameters such as concentration, electrical conductivity, surface tension, flow rate, electrical field strength, type of electrodes, and relative humidity, determine the dimensions and shape of the fiber structure. The concentration of the polymer, the solvent system [47,48] (i.e., type of solvent or relative ratio in the binary solvent system), and ambient humidity play significant roles in causing the shape change from bead to fiber. Additionally, the secondary surface morphology of the fiber is regulated by changes in the applied voltage, flow rate, and distance between the needle tip and collector (DTC), which can result in different fiber structures such as porous, grooved, smooth, and rough fibers [49,50,51]. Similarly, other experimental conditions affect the crystallinity and fraction of β-phase properties of PVDF nanofibers [52], requiring the precise control of conditions to obtain samples with optimal harvesting properties. It was found that the β-phase content increased when using a solvent with a low boiling point or under a low process temperature, and with a decreasing DTC and flow rate [53]. Therefore, this section focuses on comparing and analyzing the properties of PVDF-based nanofibers under various experimental conditions during electrospinning.

### 2.1. PVDF

**Figure 5:**
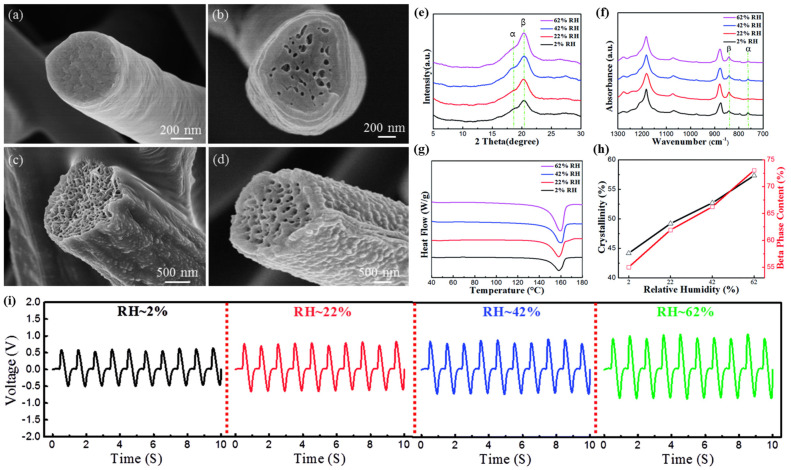
SEM image of PVDF nanofiber according to humidity (**a**) 2%, (**b**) 22%, (**c**) 42%, (**d**) 62%, (**e**) XRD, (**f**) FT-IR spectra, (**g**) DSC data, (**h**) relationship between ΔXc and F(β), and (**i**) output voltage of PVDF mat at different levels of RH (2, 22, 42, 62%); Reproduced with permission from ref. [54]; Copyright 2018, Royal Society of Chemistry.

Zaarour et al. [52]. compared the changes in the fiber morphology of PVDF at different relative humidity levels and investigated the correlation between humidity, crystallinity, and piezoelectric properties. As the relative humidity increased, the smooth fibers gradually transformed into highly rough and porous fibers, and the average diameter of the fibers tended to increase from approximately 836 nm at 2% RH to approximately 2698 nm at 62% RH (Figure 5a–d). In addition, the β-phase fraction and degree of crystallinity of PVDF measured from XRD patterns (Figure 5e–h), FT-IR spectra, and DSC data tended to increase as the humidity increased (Figure 5i), which was interpreted as a phenomenon caused by the increased evaporation time during the electrospinning process. Consequently, the β-phase fraction corresponded to the piezoelectric output, establishing a positive linear relationship between the relative humidity and the piezoelectric properties of the PVDF nanofiber webs [54].

Hu et al. conducted a study on the established relational expression between the phase structure and piezoelectric outputs to effectively predict the conversion of mechanical energy into electrical energy in PVDF fiber devices. The influence of PVDF fiber on the electric field and the rotating speed of the collector was investigated, and the degrees of crystallinity and β-phase proportion under each condition were measured. Based on these data, the piezoelectric outputs of the final devices were correlated, and the maximal outputs of 2.8 V and 1.32 μA were found in the optimized parameters at 15 kV and 500 rpm. In addition, the correlation between the content effective piezoelectric phase, which means the actual content of the β-phase, and the piezoelectric response was established. Finally, a linear dependence between the piezoelectric outputs and the effective piezoelectric phase of the devices was proposed [39,55,56].

Ribeiro et al. studied the influence of processing conditions such as applied voltage, needle diameter, and flow rate on the morphology and polymorphism of electrospun PVDF membranes. In the condition with high applied voltage, higher elongation forces are exerted due to the higher charge density induced on the surface of the jets, leading to a gradual decrease in the diameter of the final fibers as the applied voltage increases. However, the clear effect of the applied voltage is a subject of discussion because changes in the applied voltage affect other parameters, such as the traveling time of the jets. The increase in the applied voltage intensified the degree of instability of the jets, resulting in a broader distribution of fiber diameters. In addition, the electroactive β-phase content decreased slightly with an increase in applied electrical voltage. However, when a larger needle diameter was used, the fiber diameter and β-phase fraction decreased significantly. Under the same conditions, the fiber diameter tended to increase slightly with increasing flow rate, and the β-phase fraction tended to increase slightly from an almost constant level. These changes are attributed to the increase in jet stretching as a function of both needle diameter and flow rate, which favors the ordering of the polymer chain into all-trans conformations [57,58].

Gee et al. studied the influence of the fraction of acetone, DTC, flow rate, and various solvents (dimethylformamide (DMF), dimethyl sulfoxide (DMSO), and *N*-methyl-2-pyrrolidone (NMP)) on the piezoelectric properties of PVDF nanofiber membranes. The highest mean β-phase fraction was observed at the largest fraction of acetone (60 v% DMF/40 v% acetone), and a decreasing trend was observed as the fraction of DMF increased. In addition, the highest mean β-phase fraction was observed at the longest DTC (16 cm) and fastest flow rate (0.8 mL/h), respectively, and the β-phase fraction increased with increasing DTC. However, a linear relationship with flow rate was not established because inconsistent fiber formation at a lower flow rate caused variation in the β-phase fraction. Among these factors, the most significant contributor to β-phase formation was observed to be the fraction of acetone, which determines the volatility of the solution and is involved in solvent evaporation during the deposition of nanofibers. Therefore, utilizing a co-solvent system with high volatility is an effective way to induce β-phase through faster solvent evaporation effectively. However, excessive solvent volatility is undesirable because it can cause clogging at the needle tip, which interferes with electrospinning. However, in an experiment comparing the piezoelectric properties of electrospun PVDF membranes using NMP or DMSO as a substitute for DMF, the highest β-phase fraction was obtained in the 60 v% DMF/40 v% acetone condition, and the lowest β-phase fraction was obtained using NMP. In addition, the relatively high density and boiling point of DMSO compared to other solvents are factors that increase the nanofiber diameter; therefore, the highest average nanofiber diameter of approximately 624 nm was observed using DMSO [59,60,61].

**Figure 6:**
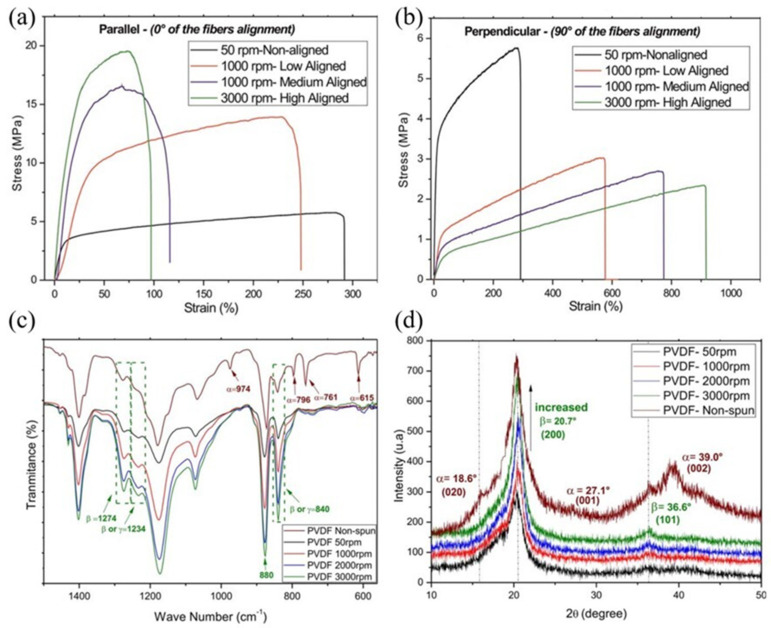
Stress–strain curve of electrospun PVDF-based device with different rotating-drum speeds; (**a**) parallel, (**b**) perpendicular. (**c**) FT-IR, (**d**) XRD spectra of non-spun and electrospun PVDF fiber with different rotating-drum speeds (50, 1000, and 3000 rpm); Reproduced with permission from ref. [62]; Copyright 2017, John Wiley & Sons.

Lins et al. conducted a study on the influence of fiber anisotropy and properties under the condition of high collector rotating speed. The centrifugal force generated by the rotating drum and the shear force generated by the jet during electrospinning can effectively induce fiber alignment in the uniaxial direction. Due to the fiber elongation caused by the centrifugal force, a decrease in the fiber diameter was observed as the rotating speed increased. When parallel stress was applied to the fiber alignment direction, the highly aligned fiber exhibited more than three times the stress at failure compared to the non-aligned fiber, which was observed to have a tensile modulus of 98.3 MPa and a stress at failure of 19.5 MPa at 3000 rpm (Figure 6a,b). Meanwhile, Fourier-transform infrared (FT-IR) absorption data suggest that the absorption peaks at 615, 761, 796, and 974 cm^−1^, corresponding to the α-phase, which are clearly seen in non-spun fibers, disappear through the electrospinning process, and new bands at 840, 1234, and 1274 cm^−1^, corresponding to the β or γ-phase, emerge. In the electrospinning process, the Coulombic force causes the alignment of the molecular chains—it promotes the formation of the β-phase, showing that the resulting non-polar α-phase is converted into the polar β-phase. Furthermore, the high-speed collector is directly related to the enhancement of the β-phase. Similar to the FT-IR data, the XRD spectra also showed that the absorption intensity of the peaks corresponding to the β-phase tended to increase with increasing rotating speed (Figure 6c,d) [62,63,64,65].

Liang et al. studied the influence of heat treatment on the mechanical properties of electrospun PVDF fibrous membranes. Experiments were designed at temperatures near the melting temperature of PVDF powder (150–160 °C), and it was observed that the average fiber diameter increased with increasing heat-treatment temperature, forming a wider distribution of fiber diameter. Additionally, under conditions below the melting point of approximately 160 °C (up to 155 °C), it was observed that the α-peaks in the WAXD pattern tended to decrease while the β-peaks increased with increasing temperature. As a result, heat treatment not only increased the fiber diameter of the PVDF membrane but also improved the inter-fiber bonding and crystallinity. This increase in fiber diameter acts as a mechanism to enhance the tensile strength of the PVDF membrane, resulting in improved tensile strength, whereas enhanced inter-fiber bonding makes it more difficult for the fibers to move, resulting in rigid properties. Because higher crystallinity enhances tensile strength and modulus, improved mechanical properties were observed with increasing heat-treatment temperature [66,67].

### 2.2. Co-Polymer

**Figure 7:**
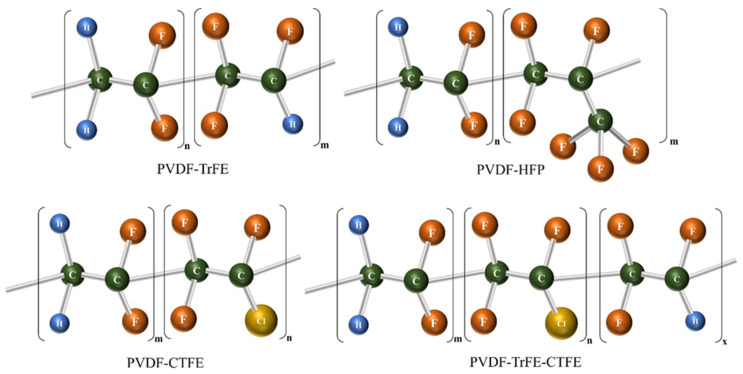
Chemical formula diagram for PVDF co-polymers.

Fluoropolymers are niche polymers used as high-value materials for high-tech applications in industries such as aerospace, electronics, coatings, membranes, cables, and automotive. Among them, VDF- and TrFE-based co-polymers exhibit remarkable electroactive properties and can be incorporated into a wide range of devices, such as printed memories, sensors, actuators, artificial muscles, and energy storage devices [68]. PVDF is a semi-crystalline polymer with interesting electroactive properties; however, typical melting and solution processing techniques result in a thermodynamically favored non-polar α-phase. In comparison, P(VDF-TrFE, PT) crystallizes directly from the polar phase under the same conditions as PVDF. In this study, blended films containing PVDF and P(VDF-TrFE) were prepared using a solvent casting method. The differences in crystallization behavior between PVDF, P(VDF-TrFE), and the resulting blended films were comprehensively investigated. The substitution of fluoride atoms in the TrFE monomer induces strong steric hindrance that can alter the crystallization process, making it more favorable for the nucleation of the PVDF β-phase. Poly(vinylidene fluoride) (PVDF) and its co-polymers possess excellent ferroelectric and piezoelectric properties and are expected to be utilized as sensors in applications such as smart wearables, hydrophones, speakers, and medical ultrasound devices. In addition to the crystalline phase, co-polymers with TrFE and TFE have been extensively studied in both academia and industry. To explain how steric hindrance between VDF and TrFE/TFE affects the condensed-state structure and electrical properties of the polymers, the electrical properties of PVDF, P(VDF-TrFE), and P(VDF-TFE) were compared. Poly(vinylidene fluoride) (PVDF)-based diastolic ferroelectric polymers show great promise for applications in transducers, sensors, and artificial muscles due to their excellent electrical distortion properties. Currently, all-trans-chain conformations of diastereomers must be stabilized by special monomers, such as TrFE or TFE units, whose dipole moments are lower than those of VDF, resulting in reduced dielectric and electrostatic properties. The direct stretching of PVDF co-polymers with bulky units, such as P(VDF-CTFE) and P(VDF-HFP), has been shown to be a viable alternative to stretching PVDF co-polymers with CTFE and HFP units. In the current work, a unique strategy for uniaxially stretching a family of PVDF polymers tuned by CF2CH bonds with a smaller steric bulk is reported to realize tunable ferroelectric performance for piezoelectric and electrochemical applications [69,70,71]. Figure 7 shows the chemical structures of PVDF co-polymers (-TrFE, -HFP, -CTFE, -TrFE-CTFE).

By modifying processing parameters such as the polymer concentration, applied voltage, feed rate, and electrospinning time/fiber mat thickness, electrospun P(VDF-TrFE) nanogenerators with a wide range of piezoelectric performances and physicochemical properties were fabricated. An artificial neural network (ANN) model was developed to estimate and predict the relationships between the processing parameters, piezoelectric performance, and fiber morphology. The results of the developed ANN model showed good agreement with the experimental results, with an error of less than 5%, indicating good potential for modeling the physicochemical properties of nanogenerators to predict untested conditions [72].

Electrical potential plays an important role in tissue engineering and wound healing. Piezoelectric nanogenerators based on the direct piezoelectric effect have attracted considerable attention because they can be used as self-powered energy sources for electrical stimulation. However, the accuracy of the piezoelectric stimulation of piezoelectric polymeric membranes in vitro under dynamic conditions has rarely been studied. In this study, a self-powered tunable electrical stimulation system was developed to aid the proliferation of osteoblasts using a well-aligned P(VDF-TrFE) piezoelectric nanofiber membrane (NFM) as a nanogenerator and scaffold. The effects of different post-treatments (annealing or poling) on the surface wettability, piezoelectric β-phase, and ferroelectric properties of the electrospun NFMs were evaluated here. The polar P(VDF-TrFE) NFMs provided improved piezoelectric values (d31 = 22.88 pC/N) and exhibited superior sensing performance compared to the initial P(VDF-TrFE) NFMs (d31 = 0.03 pC/N). The maximum voltage and current output of the P(VDF-TrFE) nanofiber PENGs reached −1.7 V and 41.5 nA, respectively. By fixing the NGs to the flexible bottom of the culture plate, accurate electrical responses were obtained in real time under dynamic mechanical stimulation, thereby replicating the actual scenario of providing electrical stimulation to the cells in vitro. In addition, the interaction between the piezoelectric nanofiber NGs and the cells was evaluated using an equivalent circuit model [73].

Piezoelectric P(VDF-TrFE) nanofibers prepared by electrospinning have attracted increasing attention in the field of flexible sensors and nanogenerators. However, the orientation dependence of the piezoelectricity of electrospun nanofibers remains elusive. In this study, the piezoelectric performance of individual nanofibers is characterized by piezoresponse force microscopy (PFM), and the effect of annealing on the β-phase crystallinity is investigated by XRD and FT-IR spectroscopy. The experimental results show that as-spun P(VDF-TrFE) nanofibers form a higher β-phase content compared to spin-coated films, and the β-phase content is increased by annealing. The annealed P(VDF-TrFE) nanofibers exhibit distinct vertically polarized switching characteristics. The high piezoelectric output in the thickness direction and the low piezoelectric output in the longitudinal direction of the nanofiber mat further confirmed that the preferential dipole orientation of the electrospun P(VDF-TrFE) nanofibers was perpendicular to the substrate surface. Highly aligned P(VDF-TrFE) nanofibers can sense orientation strain owing to their piezoelectric and mechanical anisotropy [41].

To improve the piezoelectric properties of PVDF-HFP, composites were prepared by adding varying amounts of AgNWs to the solutions of PVDF-HFP and DMF films. Tensile testing and poling were applied to the films to induce polarized β-phase formation and dipole moment reorientation. The crystal structures of the films were investigated using polarized optical microscopy (POM), FT-IR spectroscopy, and DSC. The results showed that the phase transition mainly occurred during stretching, and the reorientation of the dipole moment was attributed to poling. It was also concluded that AgNWs act as nucleating agents in β-phase formation and phase transformation. Piezoelectric properties were evaluated based on the output voltage and harvested power density. The open-circuit voltage of the film containing 0.1 wt% AgNWs was 52% higher than that of the pure PVDF-HFP film. In addition, the harvested power density increased by 159%. Overdosing with AgNWs leads to crystal defects and low crystallinity, which deteriorate the piezoelectric properties [74].

**Figure 8:**
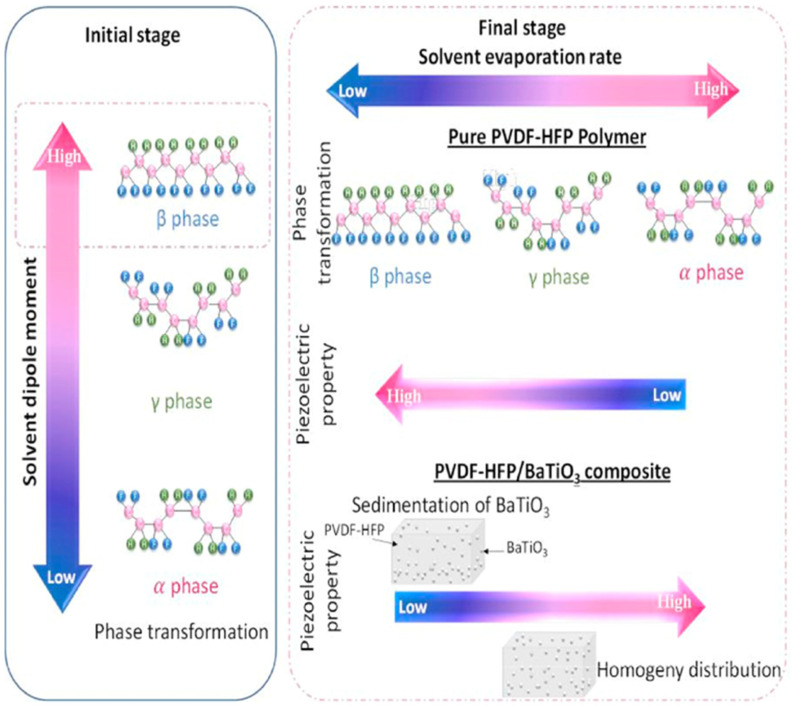
The impact of solvent dipole moment and evaporation rate on the crystalline transition and piezoelectric characteristics of pristine PVDF-HFP and PVDF-HFP/BaTiO_3_ blends); Reproduced with permission from ref. [75]; Copyright 2021, Elsevier.

In this study, an approach for developing a high-performance flexible nanogenerator based on a hybrid piezoelectric composite is reported. Figure 8 shows how the solvent dipole moment and evaporation rate affect the crystalline transition of PVDF-HFP in this study. The approach involves first using solution-mixing methods with different solvents to determine a suitable solvent to achieve better piezoelectric properties of piezoelectric ceramic polymer-composite materials. Conductive silver nanoparticles were incorporated to improve the performance of the nanogenerator. Using scanning electron microscopy (SEM), XRD, and FT-IR spectroscopy, different aspects were considered, namely the homogeneity of the particle distribution in PVDF-HFP and the crystallinity of the composites. This study demonstrates the efficiency of DMF in increasing the crystalline phase transition owing to its high dipole moment, which leads to a suitable evaporation rate and improved piezoelectric performance. This approach explicitly demonstrates the effectiveness of doping with Ag nanoparticles (NPs) to enhance piezoelectric performance. The composites exhibit an enhanced output voltage of approximately 2.21 V and output power of 0.22 μW, which are approximately three and nine times higher, respectively, than the composites without Ag NPs. The NGs also exhibited excellent stability over 900 cycles, indicating their robustness. This approach extends the performance limits of the PVDF-HFP-based NGs and their potential applications. Bouhamed also demonstrated the feasibility of using optimized NGs to harvest mechanical energy from human activities, with the ability to generate approximately 3.56 V by tapping with the palm [75].

## 3. Applications of PVDF Mesh to Nanogenerator

PVDF is a particularly attractive piezoelectric material because of its excellent properties. It has been demonstrated that the piezoelectric properties of PVDF can be enhanced by electrospinning. In recent years, there have been numerous technological innovations in various aspects such as the capture equipment and pretreatment of electrospun PVDF nanofibers. First, this article briefly introduces electrospinning. In the second section, we review recent research on the preparation of electrospun PVDF nanofibers. Research on the promising applications of electrospun PVDF nanofibers includes biomedicine, sensors, and energy harvesting due to their flexibility, biocompatibility, and excellent performance [76,77,78].

Long-term observation of the triboelectric effect has not only demonstrated the feasibility of many new and useful triboelectric devices (triboelectric nanogenerators) but has also continued to motivate the exploration of its mysterious nature. In pursuit of a comprehensive understanding of the triboelectric process, a more accurate description of the triboelectric effect and its associated parameters and factors is required. This review critically examines the basic theories and fundamental principles governing triboelectric processes; examines the differences in each charged medium; discusses electron, ion, and mass transport; and provides theoretical reasoning over the past few decades. Using information from the triboelectric series, interesting phenomena including cyclic triboelectric sequences and asymmetric triboelectricity were analyzed in detail. The interaction between the triboelectric system and its operating environment was then analyzed, and the basic explanations for the triboelectric process and its impact on the results were summarized. Triboelectric nanogenerators (TENGs) are promising candidates for powering portable wearable devices. For example, TENGs have demonstrated flexibility, a light weight, biocompatibility, versatility, and good performance. Textiles are potential substrates that wearable technologies are increasingly integrating; however, power delivery remains an ongoing challenge. TENGs are potential fiber-based mechanical-energy-harvesting power supplies, and efforts have been made to combine TENGs with textiles. There are significant challenges in integrating TENGs without losing their performance or the original properties (appearance, breathability, washability, and durability) and the feel of the fabric. Various approaches have been demonstrated to fabricate textile-based TENGs (T-TENGs). Based on their structure, T-TENGs can be classified into two main types: fabric- and fiber-based. Fabric-based TENGs consist of conventional and/or modified fabrics that act as the substrates and triboelectric materials, respectively. Material selection is critical for TENGs because the triboelectric effect of materials is fundamental. Several parameters such as power density, stability, flexibility, and sustainability must be considered when designing a TENG for a specific application. This critical review summarizes and evaluates the material selection for TENGs in a variety of applications [77,78,79,80].

### 3.1. Piezoelectric Nanogenerator (PENG)

Chansaengsri et al. fabricated a PENG using microfibrous composites composed of PVDF and PVDF with barium titanate nanoparticles. For effective, low-cost, and stable PENGs, paper and silver ink were used to fabricate paper-based integral circuit devices. The maximum open-circuit voltage and maximum short-circuit current of the electronic PENG based on PVDF were 13 mV and 380 nA, respectively. The results for PVDFr/BaTiO_3_ were 528 mV and 661 nA, respectively [81].

**Figure 9:**
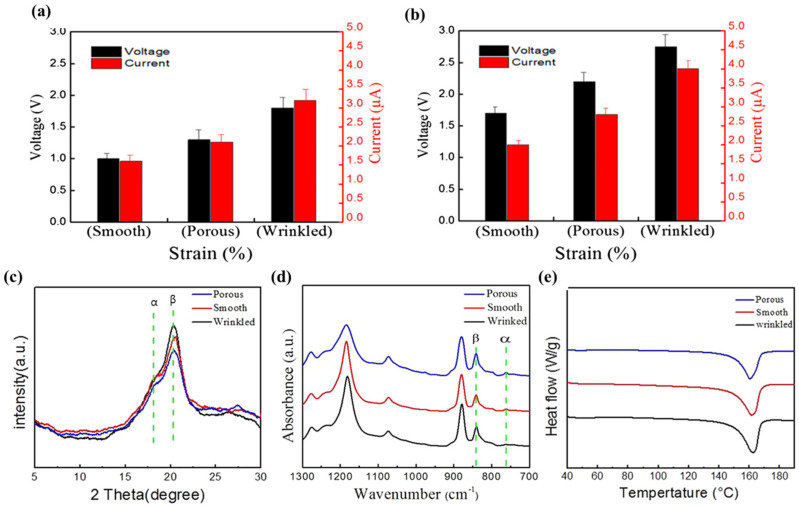
Average voltage and current of three types (smooth, porous, wrinkled) (**a**) randomly oriented PENG fiber (**b**) aligned PENG fiber. (**c**) XRD pattern (**d**) FTIR spectra (**e**) DSC data of three types (smooth, porous, wrinkled) aligned PVDF fiber; Reproduced with permission from ref. [82]; Copyright 2019, John Wiley & Sons.

Zaarour et al. investigated the relationship between the surface morphologies (wrinkled, smooth, and porous) and electrical outputs of the PENG based on randomly oriented and aligned electrospun fibers. The experimental results showed that the output voltage and current of the PENG based on random fibers were lower than those based on aligned fibers. In addition, the results were in the order of smooth < porous < wrinkled (Figure 9a,b). The voltage and current outputs of the PENG based on the randomly oriented smooth-fiber web were approximately 1 V and 1.6 μA, while the voltage and current outputs of the PENG based on the aligned wrinkled-fiber web were approximately 2.8 V and approximately 3.9 μA. Moreover, the difference in intensity at 2θ = 20.6° peak representing the β-phase was evident when examining the crystalline phase of the XRD (Figure 9c). Similar to the output voltage/current, the β-phase content of aligned fibers was higher than that of randomly oriented fibers in the order wrinkled > smooth > porous. Table 1 shows the numerical measurements of β-phase contents and crystallinity according to the structure of the fibers [82].

Khalifa et al. fabricated flexible piezoelectric PVDF/g-C_3_N_4_ nanocomposite fibers (PGN-X) by adding graphitic carbon nitride (g-C_3_N_4_) to increase the spinnability and β-phase content of nanofibers and studied them for a nanogenerator that realizes energy harvesting through human hand movements (tapping, thumb imparting, palm imparting, bending). The tensile strength increased as the content of g-C_3_N_4_ increased but tended to decrease at 0.5 wt% and above. The PGN-0.75 sample showed the highest piezoelectric voltage of approximately 7.5 V and 0.23 μA performance, with excellent stability and no decrease in piezoelectric performance after 18 weeks [83].

Mansouri et al. fabricated electrospun PENGs with different ratios of DMF and THF (1:1 and 3:1), different proportions of PVDF (15 and 18 wt%), and different proportions of ZnO additives to enhance the piezoelectric effect. The effective output power was calculated using the peak-to-peak values of the output current and voltage of the root mean square (RMS) of the piezoelectric nanogenerators, and the RMS output voltage of the fiber samples increased when the frequency was increased. Furthermore, the increase was greater for samples with higher levels of zinc oxide, with a maximum output power of 32 n/W/cm^2^. However, the electrosprayed films with lower PVDF concentrations exhibited a decrease in output voltage [84].

**Figure 10:**
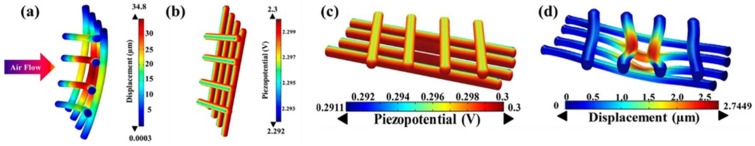
Distribution of NFs’ (**a**) displacement (**b**) piezopotential during air flowing and distribution of NFs’ (**c**) displacement (**d**) piezopotential during human mouth blowing; Reproduced with permission from ref. [85]; Copyright 2018, ACS Publications.

Alam et al. prepared ZnO-containing paper ash (ZPA) in a simple and fast manner and added it to PVDF nanofibers to increase the amount of the electroactive β-phase to produce superior PENGs. In this study, the piezopotential of PVDF nanofibers was measured in situ during air flow (Figure 10). The reason for adding ZPA is its β-phase content of 92%, which is superior to other additives (ZnO-87%, BTO-80%, CNTs-84%). A nanofiber nanogenerator (NFNG) based on PVDF was realized for energy harvesting using wind, and the maximum output voltage was 4.8 V under 145 Pa of wind. In addition, the NFNG can harvest at least 1 V of energy when wind is blown by the human mouth [85].

Alam et al. fabricated a nanogenerator (NG) with nanoparticle TiO_2_, which plays an important role as an external filler in PVDF, to increase the piezoelectric β-phase by 16% and mechanical properties by 148% (tensile strength). The NG was used to realize energy harvesting using human motion and acoustic sensitivity. The output voltages of TiO_2_ doped PVDF NGs ranged from 1.2 to 2.8 V depending on human motion (swimming motion of legs, walking, and running). In addition, an outstanding electrical output (17.5 V) was realized by NGs under acoustic vibrations. This indicates that NGs can serve as tiny electronic devices to monitor noise pollution or operate in a self-powered mode [86].

He et al. fabricated a wearable self-powered electrochromic supercapacitor (ESC) with a PVA/H_2_SO_4_ gel electrode and PVDF-based energy-harvesting PENGs in this experiment. Patterned PANI electrodes were used in the ESC to serve as energy storage and indication devices, and PVDF nanofiber-based PENGs were used to harvest the mechanical kinetic energy of the human body to realize a self-charging, self-powered system. Experiments with PENGs attached to a human body produced up to approximately 0.3 V [87].

Sun et al. fabricated a PVDF-ZnO-based piezoelectric acoustoelectric nanogenerator with a hierarchical microstructure using electrospinning and hydrothermal techniques. It could produce up to 1.12 V and 1.6 μA in a 140 Hz and 116 dB sound environment. In addition, under optimized sound conditions, the PVDF-ZnO acoustoelectric nanogenerator could charge a capacitor of 1.3 V in 3 min [88].

### 3.2. Triboelectric Nanogenerator (TENG)

Hao fabricated a triboelectric nanogenerator (TENG) based on ES PVDF nanofiber. In the fabrication of the TENG, a high output voltage and power were obtained by controlling the electropositive material, solvent ratio, friction angle, and polymer humidity. The TENG built with a PVDF mat and PHBA showed the highest result of −112/+88 V, and the highest result was obtained when the ratio of DMF was 60 wt%. In addition, −119/+105 V was obtained when the friction angle of PVDF and PHBV nanofiber mats was 90°, and the lower the polymer humidity, the higher the obtained result [89].

Yu et al. fabricated PVDF nanofibers with a biomimetic water–hyacinth petiole (BWHP) structure to produce BN-TENG with a honeycomb mold pattern. This BWHP nanofiber structure is simple, inexpensive, and has a positive effect on the surface charge density of triboelectric materials. As the volume ratio of mineral oil in the PVDF solution increases, the output voltages initially increase and then decrease, reaching a maximum of 670 V and 55 μA at a volume ratio of 0.3 [90].

Garcia investigated TENGs utilizing the electronic properties of PVDF and PVP, rather than the piezoelectric properties of PVDF, and observed the relationship between the applied physical stimulus and electrical response in real time using a dynamic mechanical analyzer. The output voltage increased as the pressure (Pa) increased and saturated at 13.1 V at a pressure of 1400 Pa or higher. The TENG outperformed the insufficient output voltage of the piezoelectric effect due to the α-phase of PVDF, which was enhanced by the strong electron-exchange ability between PVDF and PVP fiber due to its large surface area [91].

Qin et al. fabricated a wearable and flexible TENG based on crumpled PVDF-HFP nanofiber membranes. The TENG utilized a unique three-dimensional corrugated structure to significantly improve the stability and stretchability of the electrical output. The device exhibited a maximum output voltage and current of 80 V and 1.67 μA with 150% tensile strain due to the corrugated structure. Using this performance, a wearable device was fabricated to drive a green light-emitting diode (LED) and monitor human movements directly on the skin [92].

Kim et al. fabricated a wearable single-electrode TENG with 80% transparency in the 550 nm light wavelength band using the Ag nanowire/P(VDF-TrFE) ES method. During the ES process of P(VDF-TrFE), the oxygen-containing functional groups on the surface of AgNWs increased the crystalline β-phase, and the F-rich surface with high electronegativity enabled efficient triboelectricification. These effects resulted in an output power density of up to 217 W/m^2^ through repeated contact and the removal of latex gloves, driving 45 LEDs with excellent performance [93].

**Figure 11:**
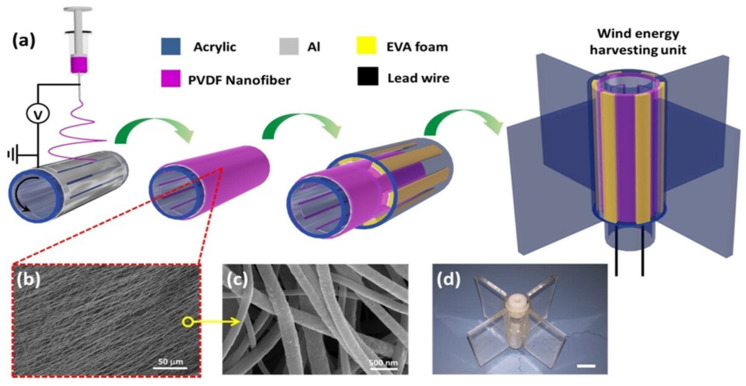
(**a**) Schematic of electrospinning and fabrication of CRF-TENG. (**b**) SEM image of PVDF nanofiber of CRF-TENG. (**c**) Enlarged image of SEM. (**d**) Photograph of CRF-TENG; Reproduced with permission from ref. [94]; Copyright 2018, Elsevier.

Ren et al. fabricated a coaxial rotatory freestanding TENG (CRF-TENG) using PVDF as the TENG material and realized wind energy harvesting, shown in Figure 11. The electrospun PVDF nanofiber membrane exhibited excellent power output characteristics and durable energy-harvesting performance owing to its rough surface and resistance to fracture. The generated electricity can be used to produce hydrogen via environmentally friendly water electrolysis. It showed a hydrogen production rate of up to 6.9685 µL/min in 1 M KOH solution at a wind speed of 10 m/s, and a maximum 650 V open-circuit amplitude, 50 μA short-circuit current, and 10 mW output power at a rotational speed of 900 r/min [94].

### 3.3. Hybrid Nanogenerator (HNG)

Here, a hybrid energy-scavenging device for in vivo applications is presented. This hybrid device consists of a piezoelectric PVDF nanofiber nanogenerator for harvesting mechanical energy, such as breathing or heartbeats, and a flexible enzymatic biofuel cell for harvesting biochemical (glucose/O_2_) energy from biofluids. These two types of energy can be used in vivo. The two energy-harvesting approaches can operate simultaneously or separately to increase output and lifetime. A hybrid device is used to power a ZnO nanowire UV light sensor to demonstrate a “self-powering” nanosystem [95].

Fuh et al. developed a fully packaged, self-powered sensor based on electrospun PVDF nanofibers. A graphene-based piezoelectric generator (GBPG) and a graphene-PET triboelectric generator (GPTG) were used in the sensor, which exhibited a performance of up to 6 V/280 nA under a physical stress of approximately 5 MPa. In addition, an output power of approximately 172 nW was achieved by a single-hand press-and-release action, and it can be self-powered by human movements (breathing, drinking, and eating). The device can be attached to clothes to act as a self-powered sensor to detect wind speeds from 0 to 11.3 m/s [96].

Guo et al. fabricated a hybrid triboelectric-piezoelectric nanogenerator (TPNG) using electrospun PVDF nanofibers. Two working modes, “separation” and “integration,” were utilized through physical motion, and the piezoelectric-enhanced triboelectricity effect was realized. Because it is a fiber composed of a combination of conductive fabric–silk fibroin fibers and PVDF fiber–conductive fabric, it provides a flexible appearance and good air permeability and has the advantage of being custom-built into clothing of the desired size and shape. In addition, thanks to the large specific surface area and the strong ability of PVDF to donate or lose electrons, it has an output voltage of up to 500 V, short-circuit current of 12 μA, and power density of 0.31 mW/cm^2^ in the “separation working mode” [97].

Sahatiya et al. implemented a piezo-triboelectric hybrid nanogenerator connected in parallel using several layers of MoS_2_ prepared through an aqueous solution process and in situ-poled PVDF. The energy was harvested through writing, signatures, and human touch. The deposition time of the electrospun nanofibers on the MoS_2_-cellulose was tested at different times of 15, 25, and 35 min. The output voltage was the lowest under the 15 min deposition conditions, and the 25 min result was approximately twice that of the 35 min result (approximately 50 V). Finally, HNG was measured to have an open-circuit voltage of 50 V, a short-circuit current of 30 nA, and a power of 0.18 mW/cm^2^ [98].

## 4. Enhancement Techniques of Harvesting Output

### 4.1. Aligning Fiber Structure

A high-performance PENG is constructed by manufacturing a multilayer piezoelectric composite material with a porous structure based on highly oriented Pb(Zr_0.52_Ti_0.48_)O_3_/PVDF (PZT/PVDF) electrospun fibers using a laminating method. PZT particles as a piezoelectric reinforcing phase are embedded in PVDF fibers, promoting the formation of the polar β-phase in PVDF. The multilayer porous structure effectively promoted overall polarization and surface-bound charge density, providing highly efficient electromechanical conversion. The PENG based on 10 wt% PZT/PVDF composite fiber with a film thickness of 220 μm outputs an optimal voltage of 62.0 V and power of 136.9 μW, which are 3.4 and 6.5 times those of the PENG based on 10 wt% PZT/PVDF cast film. Importantly, the PENG shows a high sensitivity of 12.4 V·N^−1^, providing significant advantages compared to PENGs with other porous structures. Additionally, the composite material exhibited excellent flexibility, with a Young’s modulus of 227.2 MPa and an elongation of 262.3%. This study demonstrates the significant potential of piezoelectric fiber composites in flexible energy-harvesting devices [99].

**Figure 12:**
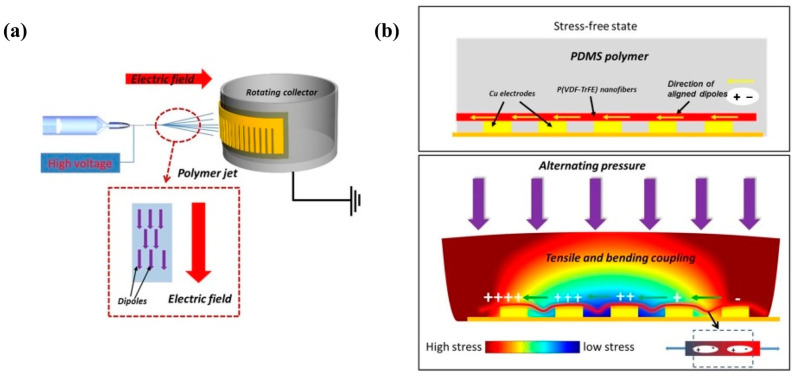
Schematic of (**a**) electrospinning process on rotating collector and (**b**) cross-section view of electrospun P(VDF-TrFE)-based nanogenerator; Reproduced with permission from ref. [100]; Copyright 2019, MDPI.

Aligned P(VDF-TrFE) nanofibers were successfully fabricated using advanced electrospinning. The ordered nature of the nanofibers was achieved using parallel electrodes and rotating-drum collectors fabricated via lithography and wet etching (Figure 12). SEM images showed that the nanofibers were highly aligned with smooth surfaces and uniform diameters. XRD and FT-IR tests indicate that the fibers contain a high β-phase content. Nanogenerators based on the aligned P(VDF-TrFE) nanofibers exhibited excellent electrical performance, with a maximum output voltage of 12 V and a peak-to-peak short-circuit current of approximately 150 nA, suggesting the possibility of applying P(VDF-TrFE) in self-powered and wearable devices [100].

### 4.2. Doping Additives

Depending on what doping additives are added to electrospinning, the performance can be improved. Many studies have been conducted on doping agents in PVDF-based electrospinnig. Pascariu’s research team present a new graphene and titanium dioxide-polyvinylidene fluoride (PVDF)-based nanocomposite (G-TiO_2_-PVDF) manufactured by the electrospinning technique and its properties. These new composite materials exhibited enhanced properties and can be used in a wide range of applications, including triboelectronics [101].

The morphology, structure, and piezoelectric performance of polyvinylidene fluoride hexafluoropropylene (PVDF-HFP) and PVDF-HFP/Co-ZnO nanofibers prepared by electrospinning were investigated. An increase in the amount of crystalline β-phase in PVDF-HFP was observed with increasing co-doped ZnO nanofiller concentration in the PVDF-HFP matrix. The dielectric constants of pure PVDF-HFP and PVDF-HFP/2 wt% Co-ZnO nanofibers are derived to be 8 and 38, respectively. A flexible nanogenerator engineered from the polymer nanocomposite (PVDF-HFP/Co-ZnO) exhibits an output voltage as high as 2.8 V compared to the pure PVDF-HFP sample (approximately 120 mV). These results indicate that the investigated nanocomposites are suitable for fabricating various flexible and wearable self-powered electrical devices and systems [102].

**Figure 13:**
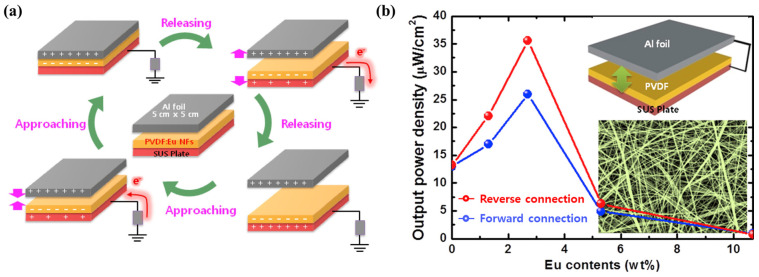
(**a**) Schematic of operating process and (**b**) relationship between output power density and Eu contents of Eu-doped PVDF-based TENG; Reproduced with permission from ref. [103]; Copyright 2018, Elsevier.

Eu-doped PVDF NFs (nanofibers) were fabricated by electrospinning and applied as active layers of TENGs. Figure 13a shows the mechanism of electricity generation from Eu-doped TENG. Structural and optical investigations showed that Eu^3+^ was successfully doped into PVDF NFs and induced a discrete emission corresponding to electronic transitions. As the Eu content increased, the phase transformation of PVDF NF from the α-phase to the β-phase was strengthened, and the diameter decreased. These changes improved the electrical output of the TENG. However, the further addition of Eu caused NO_3_-related complexes to precipitate on the surface of the PVDF NFs, which was detrimental to the performance of the TENG. Due to these trade-off effects, the output power increased from 13 to 26 μW/cm^2^ as the Eu content increased from 0 to 2.7 wt%, while it significantly decreased to 4.9 μW/cm^2^ when the Eu content further increased to 5.3 wt% (Figure 13b). Therefore, an optimal Eu doping amount has a beneficial effect [103].

The synergistic effect of electrospinning and nano-alumina trihydrate (ATH) filler was used to enhance the electroactive phase of PVDF. Electrospun nanofibers of a PVDF/ATH nanocomposite (PANCF) were synthesized using different ATH loadings. The presence of ATH enhanced the surface charge of the electrospun droplets, leading to thinner fibers. The highest β-phase content was found to be 70.1% for PANCF with 10% ATH. The piezoelectric performances of the nanofiber mats were studied using a unique setup. The PANCF with 10% ATH produced the highest voltage output (840 mV). These nanofibers are promising materials for use in sensors, actuators, and energy-harvesting applications [104].

Methylammonium lead bromide (CH_3_NH_3_PbBr_3_) (MAPbBr) was introduced into poly(vinylidene fluoride) (PVDF) nanofibers synthesized by electrospinning. The mechanical, optical, and energy-harvesting properties of this composition, as well as the β-phase composition and crystallinity, were investigated. An enhanced electroactive phase composition (91%) and a significant improvement in the tensile strength of the PVDF nanofiber mats were observed with the addition of MAPbBr. This increase in the electroactive phase composition may have played an important role in the enhancement of the output power. Acoustic nanogenerators (ANGs) made of MAPbBr-doped PVDF nanofibers (NFs) can be utilized for energy-scavenging from acoustic vibrations with higher levels of acoustic sensitivity (approximately 13.8 V Pa^−1^) and efficiency (58.5%) [105].

In a study of electroactive β-phase enhancement and piezoelectric sensing of chitosan-doped PVDF films, chitosan was reported to promote the α-phase to β-phase transition of PVDF. The -OH and -NH_2_ groups of chitosan interacted with the H and F atoms of PVDF to increase the proportion of the β-phase, which greatly improved the piezoelectric performance of PVDF. More precisely, for PVDF/chitosan composites doped with 2 wt% chitosan, the proportion of the β-phase increased from 47.9% to 63.7%. In addition, flexible pressure sensors based on this composite exhibited a low detection limit of about 220 Pa and a wide sensing range from 220 Pa to 81 kPa [106].

Lightweight tactile sensors with multimodal capabilities such as wearability, self-powered operation, and mechanical robustness are of utmost importance for new portable electronic devices involving human–machine integration. In this study, an all-fiber wearable nanogenerator (NG) was designed using a platinum (Pt) nanoparticle (NPs) interface and highly aligned 1D poly(vinylidene fluoride) (PVDF) nanofiber arrays (Pt-PVDF NFs). It is sandwiched between flexible conductive fabrics composed of interlocking microfiber arrays. The in situ synergistic enhancement of piezoelectricity in nanodimensionally confined 1D Pt-PVDF NFs arises from the application of an extensional force to an electrified solution jet, mechanical stretching for rapid collection, and Pt-NPs interfacing with the macromolecular PVDF chains. As a result, the mechanically robust structure of Pt-PVDF NFs has a five-fold improved piezoelectric figure of merit (FoMp) of approximately 9.7 × 10. The nanogenerator (PtPENG) was demonstrated to be a high-performance (output voltage, Voc of approximately 30 V and current, Isc approximately 6 mA cm^−2^) and durable (approximately 90,000 cycles) power generation device (output power, 22 µW) [107].

Research on piezoelectric composite structures has focused on enhancing the efficiency of energy harvesting and the charge performance. Within this domain, a particular piezoelectric arrangement comprising 0.4Pb(Zn_1/3_Nb_2/3_)O_3_–0.6Pb(Zr_0.5_Ti_0.5_)O_3_, known as PZN–PZT, has been identified as superior for optimizing energy-harvesting capabilities. Different contents of approximately 54 nm PZN–PZT nanoparticles were embedded in a poly(vinylidene fluoride-trifluoroethylene) [P(VDF-TrFE)] matrix by a single electrospinning process. Single-phase perovskite nanoparticles were synthesized by combustion using polyacrylic acid (PAA) as the combustion agent. The enhanced energy-harvesting performance of the flexible composite nanogenerator was confirmed by exhibiting approximately 3.4 V output voltage and approximately 240 nA output current for a single sheet containing 20 vol% nanoparticles. The charging capacity was improved by designing the laminated nanofiber composite sheets in series, suggesting that the charging rate and saturation voltage depend on the number of composite sheets connected in series [108].

### 4.3. Coaxial Electrospinning

In this study, a highly flexible PENG was designed using polyvinylidene fluoride (PVDF) and its co-polymer, polyvinylidene hexafluoropropylene (PVDF-HFP), which incorporated two nanostructures of semiconductor metal oxides: TiO_2_ and ZnO. Nanotubes of TiO_2_ and ZnO were inserted into these different polymer media by solvent mixing, and new fiber mats were created by coaxial electrospinning. This process aligns the dipoles of polymers and nanomaterials, which is a prerequisite for higher piezopotentials. Customized lightweight fiber mats with excellent mechanical strengths and flexibilities can generate excellent output voltages (up to 14 V) at different frequencies and during various mechanical vibrations in response to human movement [109].

**Figure 14:**
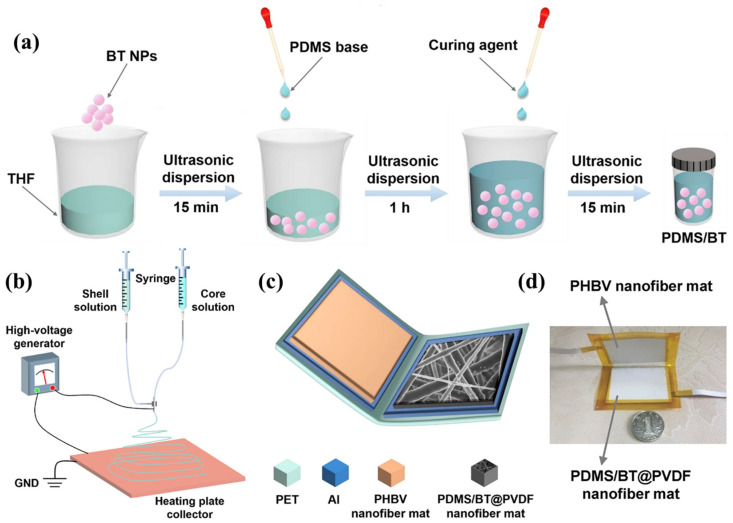
(**a**) Schematic of PDMS/barium titanate solution preparation and (**b**)coaxial electrospinning process, (**c**) TENG structure, and (**d**) digital image of BTCN-TENG; Reproduced with permission from ref. [110]; Copyright 2020, Elsevier.

Synergistic enhancement through nanoscale dielectric and dispersive modulation is reported for a barium titanate-doped coaxial nanofiber-based triboelectric nanogenerator (BTCN-TENG) (Figure 14). Polydimethylsiloxane (PDMS) and barium titanate nanoparticles (BT NPs) are combined into the core of the coaxial nanofibers instead of the existing composite film by in situ curing in the electrospinning process, and the BT NPs contained in the PDMS core layer provide the dielectric properties of the composite nanofiber mat. Constants and output performance of BTCN-TENG. Additionally, the improved dispersion of BT nanoparticles in the PDMS core layer compared to that in the PVDF shell layer was found to significantly contribute to the output performance of BTCN-TNEG. The enhanced BTCN-TENG demonstrates an elevated output voltage reaching 1020 V, along with a current output of 29 μA, and achieves a peak power density of 2.2 W/m^2^ when operating under a load resistance of 30 MΩ [110].

### 4.4. Electroless Plating

Two strategies to improve the piezoelectric sensing performance of polymer-based piezoelectric nanofibers are reported: the formation of barium titanate (BTO)/P(VDF-TrFE) composite nanofibers and the fabrication of penetrating electrodes to enlarge the interface. BTO/P(VDF-TrFE) nanofibers with a BTO weight fraction of 5 wt% exhibit maximum β-phase crystallinity and piezoelectricity. The piezoelectric output of the BTO/P(VDF-TrFE) nanofiber mats was significantly improved compared with that of pristine P(VDF-TrFE), as confirmed by PFM and compressive load tests. Oxygen (O_2_) plasma treatment was used to form the perforated electrode, followed by electroless plating. The BTO/P(VDF-TrFE) nanofibers with perforated electrodes exhibited increased dielectric constants and improved piezoelectric outputs. A sensor based on BTO/P(VDF-TrFE) nanofibers with perforated electrodes can identify energies of free-falling balls as low as 0.6 μJ and detect the movement of walking ants [111].

**Figure 15:**
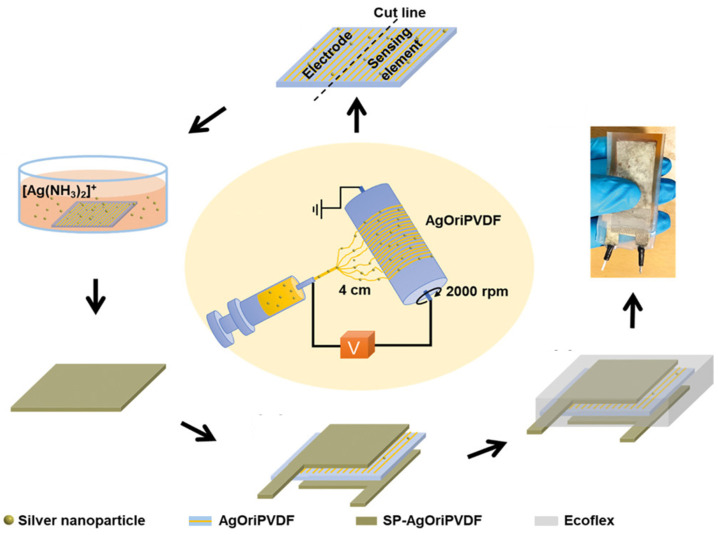
Schematic of preparation of electrospun AgOriPVDF bending sensor with SP-AgOrilPVDF electrode via ELP; Reproduced with permission from ref. [112]; Copyright 2019, ACS Publications.

A design strategy for fabricating a flexible bend sensor (BS) with ultrasensitivity to airflow using all-poly(vinylidene fluoride) (PVDF) nanofiber web-based sensing elements and electrodes for monitoring human respiration is proposed. Figure 15 shows the process of fabricating the AgOriPVDF bending sensor. A sensing element (AgOriPVDF, β-phase crystallinity approximately 44.5%) was introduced by introducing unique electrospinning (collector rotation speed of 2000 rpm and tip-collector distance of 4 cm) using silver nanoparticle interfacing. Subsequently, AgOriPVDF was transformed into a flexible and electrically conductive electrode using electroless silver (Ag)-plating technology (SP-AgOriPVDF). Interestingly, when the AgOriPVDF BS encapsulated with SP-AgOriPVDF electrodes was subjected to airflow, it demonstrated outstanding piezoelectric bending performance, achieving an open-circuit peak-to-peak output voltage (Vp-p) of approximately 4.6 V. This performance is on par with that of electrodes made from conductive tape (Vp-p of approximately 0.02 V), marking an improvement that is 200-fold more significant than the output from an unencapsulated, randomly oriented PVDF nanofiber web BS [112].

### 4.5. Modifying Structure of Device

**Figure 16:**
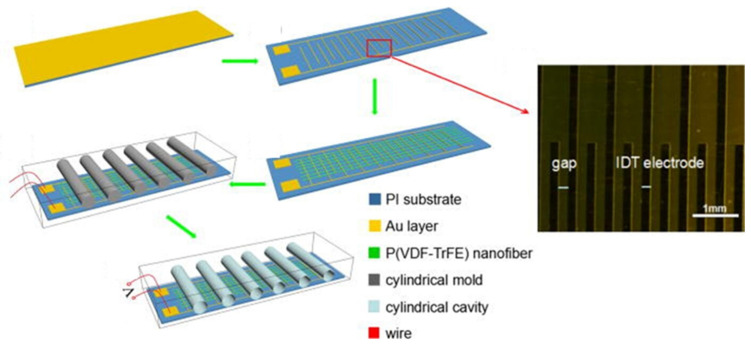
Schematic of nanogenerator fabrication process; Reproduced with permission from ref. [13]; Copyright 2018, AIP Publishing.

An Au interdigital electrode (IDT)/P(VDF-TrFE) nanofiber film based on poly(vinylidene fluoride-trifluoroethylene) [P(VDF-TrFE)] nanofibers with an aligned cylindrical cavity structure is shown in Figure 16. Piezoelectric nanogenerators were prepared by incorporating an Au IDT. A rotating collector was used to obtain highly aligned P(VDF-TrFE) nanofiber arrays. The Au IDT not only acts as a parallel electrode to collect P(VDF-TrFE) nanofibers during electrospinning but also serves as a charge collection electrode in the nanogenerator. Well-aligned cylindrical cavities enhance the deformation of P(VDF-TrFE) nanofiber films when subjected to external forces, thereby improving printing performance. The nanogenerator functioned well. As an example, Gui demonstrated energy harvesting from human walking with a peak output voltage of 5 V and a peak short-circuit current of 1.2 µA [13].

A simple yet highly efficient method for improving the output performance of piezoelectric devices containing electrospun nanofiber mats is presented. By assembling multiple nanofiber mats, Kim’s research team took advantage of the larger piezoelectric source in the spun fibers, providing improved voltage and current outputs compared to single devices. In addition to the multilayer assembly, microbead-based electrodes were integrated with nanofiber mats to deliver combined compressive and tensile force excitations to the piezoelectric layer. A vacuum-packaging process was performed to achieve a rigid and well-organized assembly of the device components, albeit with a total thickness of several millimeters. The integrated piezoelectric element showed a maximum voltage and current of 10.4 V and 2.3 μA, respectively. Additionally, the rock-solid integrity of the device components can provide high-precision sensitivity to detect small pressures down to approximately 100 Pa while maintaining a linear input–output relationship [113].

A novel yarn is presented as a wearable piezoelectric nanogenerator (PNG), consisting of a layer of electrospun poly(vinylidene fluoride) (PVDF) nanofibers integrated with a PVDF film around a silver-deposited nylon filament acting as an internal electrode. The electrospun PVDF nanofibers were wrapped around the core yarns and coated with a PVDF solution using a custom electrospinning device. The mesh of the PVDF electrospun layer ensured a uniform coating of the PVDF solution, and after coating, the PVDF film not only strengthened the wear resistance of the piezoelectric polymer layer but also improved the interface properties with the internal electrode. The resulting unique yarn produced an average peak voltage of 0.52 V, peak current of 18.76 nA, and power density of 5.54 μW cm^−3^ under a cyclic compression of 0.02 MPa at 1.85 Hz [114].

### 4.6. Other Methods

This paper demonstrates that the dimensional reduction of poly(vinylidene fluoride-trifluoroethylene) (P(VDF-TrFE)) at the nanoscale by electrospinning combined with appropriate heat treatment leads to a transformational enhancement of the piezoelectric performance. Specifically, the piezoelectric coefficient (d33) reached a maximum of −108 pm V^−1^, approaching that of inorganic materials. The electrospun mat composed of 15 μm thick heat-treated 30 nm nanofibers produced a consistent peak-to-peak voltage of 38.5 V and a power output of 74.1 μW at a strain of 0.26%, maintaining energy production through 10 k repetitive operations. Excellent piezoelectric performance was achieved through the improvement of the piezoelectric dipole alignment owing to the synergistic effect of dimensional reduction, heat treatment, and flexoelectric implementation [21].

A high-power piezoelectric energy-harvesting material was produced using hybrid PVDF/ZnO nanofibers deposited via electrospinning. During the electrospinning process, strong electric fields and stretching forces align the dipoles in the nanofiber crystals, causing the non-polar α-phase (random orientation of the dipoles) to transform into the polar β-phase in the resulting nanofibers. The effect of additional ZnO nanowires on nanofiber β-phase composition and output voltage was investigated. The maximum output voltage generated by a single hybrid PVDF-ZnO nanofiber (33 wt% ZnO nanowire) was more than 300% of the voltage generated by a single nanofiber composed of pure PVDF. ZnO NWs have been used as both a semiconductor and piezoelectric material. The electrical conductivity of the hybrid PVDF/ZnO nanofibers increased by more than four-fold when exposed to ultraviolet (UV) light [115].

A new breathable piezoelectric membrane was developed by growing zinc oxide (ZnO) nanorods on the surface of electrospun poly(vinylidene fluoride) (PVDF) nanofibers using a low-temperature hydrothermal method. Significant improvements have been made in the piezoelectric response of PVDF membranes without compromising their breathability or flexibility. The fabrication of the ZnO@PVDF porous electrospun membranes involves simple low-temperature ZnO growth in an aqueous solution that does not weaken the polarization of the resulting PVDF during electrospinning under high electric fields [116].

## 5. Further Application to Various Sensors

### 5.1. Tactile and Human Motion Sensor

Flexible nanofibers comprising BNT-ST (0.78Bi_0.5_Na_0.5_TiO_3_–0.22SrTiO_3_) and polyvinylidene fluoride-trifluoroethylene (PVDF-TrFE) were utilized in constructing a wearable piezoelectric energy harvester featuring core-shell piezoelectric structures with external electrodes. Initially, PVDF-TrFE was electrospun, and then core-shell piezoelectric nanofiber yarns were created by twisting the yarns around conductive threads. The core-shell piezoelectric nanofiber yarn was braided with conductive yarn to form the external electrode layer. Subsequently, core-shell piezoelectric nanofiber yarns with external electrodes were directly sewn into the fabric. The bending test investigated the output voltage based on the total length, effective area, and suture spacing of the piezoelectric fibers, optimizing the piezoelectric stitch pattern of the fabric. When the piezoelectric thread was sewn into the fabric, the output voltage improved with increasing pressure, and the output voltage characteristics under various body movements during bending and compression were examined [117].

**Figure 17:**
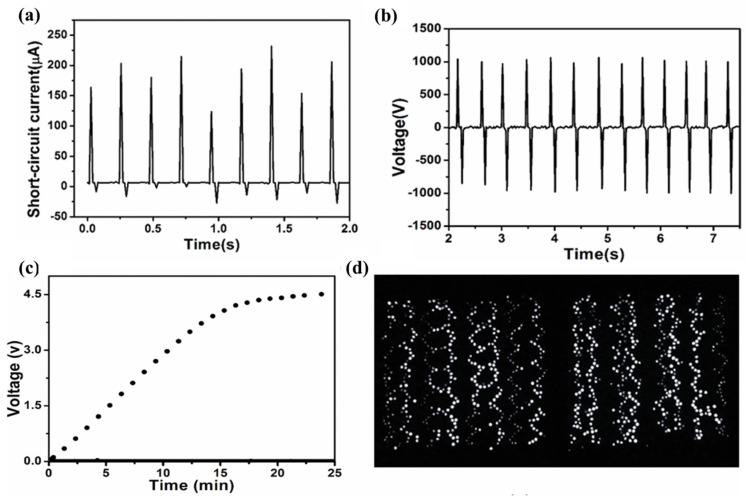
(**a**) Short-circuit current, (**b**) open-circuit voltage of PANI-based TENG under 2.5 Hz frequency. (**c**) Charging curve of 47 μF capacitor and (**d**) image of lighting up 944 LEDs by 10 × 10 cm^2^ TENG in human movements; Reproduced with permission from ref. [118]; Copyright 2019, Elsevier.

A TENG based on plain fabric utilizing polymerized polyaniline (PANI) as the electrode and polycaprolactone (PCL), to ensure a good fit between the fabric and the friction material, is presented. TENGs exhibit excellent softness and specific gas permeability, enhancing the comfort of wearable smart health-monitoring devices. The TENG’s output electricity can reach 200 µA and 1000 V at a frequency of 2.5 Hz, capable of driving approximately 1000 LEDs and providing continuous power for electronic applications (Figure 17). This truly wearable generator serves as an effective information interface for critically ill patients, and it can monitor the patient’s breathing status in real time and sound an alarm in case of breathing cessation. Meanwhile, patients facing communication difficulties can convey messages by tapping their fingers using Morse code. Importantly, experimental demonstrations show that this self-powered sensor can function normally despite additional contact resistance [118].

**Figure 18:**
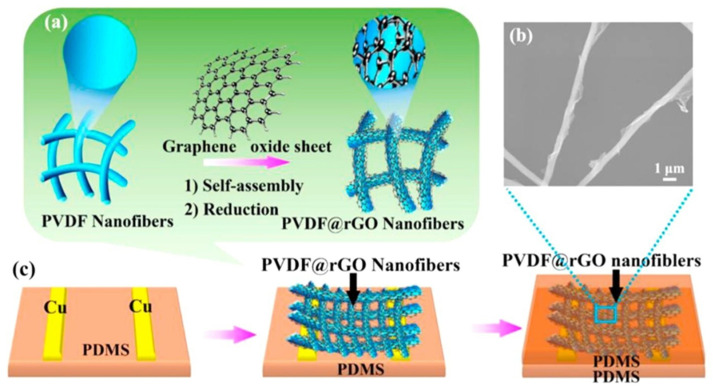
(**a**) Schematic of process where rGO nanosheets encapsulate PVDF fibers through electrostatic forces. (**b**) Electron microscopy image of PVDF@rGO nanofibers and (**c**) schematic of fabrication of flexible device; Reproduced with permission from ref. [119]; Copyright 2016, Elsevier.

Designing pressure sensors with ultrahigh sensitivity, fast response, and long-term stability is a critical step in realizing high-performance electronic skins. Herein, Lou reports the fabrication of a self-assembled 3D film platform combining the natural viscoelastic material P(VDF-TrFe) and the electrically conductive material rGO for the first time (Figure 18). Specifically, the modular assembly of rGO-encapsulated P(VDF-TrFe) nanofibers exhibits high sensitivity (15.6 kPa^−1^) and a low detection limit (1.2 Pa), and it operates at a voltage of 1 V. This platform is particularly attractive due to its excellent long-term stability of less than 100,000 cycles and a fast response time of 5 ms at 50 Hz [119].

A self-powered nanofiber-based triboelectric sensor (SNTS) was fabricated via batch-scale manufacturing technology using electrospinning and screen printing for health monitoring via respiratory monitoring. Typically, arch-structured SNTSs are assembled using nanofiber membranes and AgNP electrodes. A conductive network of nanofiber piles and Ag nanoparticles provides gas channels throughout the device. The gas permeability of SNTSs is high at 6.16 mm/s, giving them an overwhelming advantage over commonly used wearable devices made of sealed-cast films. Due to the softness of the nanofiber membrane, the SNTS exhibits excellent electronic output performance, regardless of bending, twisting, or folding [120].

### 5.2. Respiratory Monitoring

Liu fabricated a wearable self-powered active sensor based on a flexible piezoelectric nanogenerator for use in respiratory and healthcare monitoring. A flexible nanogenerator was fabricated by polarizing an electrospun poly(vinylidene fluoride) thin film onto a silicon substrate, and its electrical properties were measured. When cyclically stretched by a linear motor, the flexible piezoelectric nanogenerator produced an output open-circuit voltage and short-circuit current of up to 1.5 V and 400 nA, respectively. Through integration with elastic bandages, wearable self-powered sensors have been fabricated and used to monitor human breathing, subtle muscle movements, and speech recognition [45].

Respiratory parameters such as the respiratory rate (RR), inspiration time (tin), expiration time (tex), and their ratio (IER = tin/tex) are clinically significant for distinguishing between healthy individuals and those with respiratory conditions. In this study, a respiration-monitoring triboelectric nanogenerator (RM-TENG) is introduced, capable of accurately assessing various parameters, including IER. By analyzing the strain profiles of the nanofiber layers using digital image correlation (DIC) testing, a mathematical model was developed to quantitatively evaluate the impact of the gap distance between the two triboelectric layers in the contact area. The RM-TENG exhibits higher sensitivity to smaller gap distances ranging from 1 to 5 mm, as the high specific area of the nanofibers enables more effective contact. Optimized structural parameters enable the RM-TENG to consistently and precisely detect the aforementioned respiratory indices with outstanding detection stability over a 40 h period [121].

**Figure 19:**
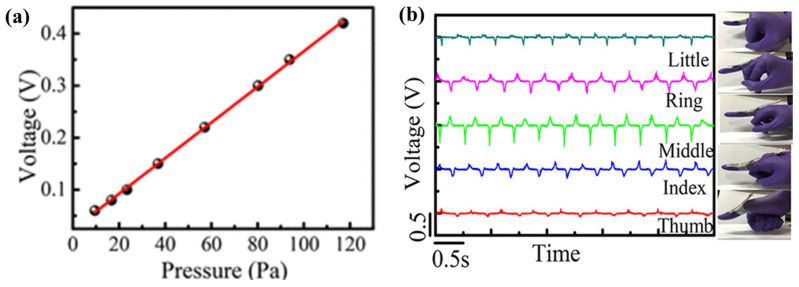
(**a**) Open circuit output voltage of graphene-based piezo and pyro-electric nanogenerator (GPPNG) according to different applied pressure. (**b**) Output voltage for each finger movement over time; Reproduced with permission from ref. [117]; Copyright 2019, ACS Publications.

A self-powered flexible piezoelectric and pyroelectric hybrid nanogenerator (NG) device designed to be affixed to multiple locations on the human skin for detecting static and dynamic pressure changes and monitoring temperature fluctuations during breathing is presented. An efficient and cost-effective manufacturing strategy was developed to produce electrospun poly(vinylidene fluoride) (PVDF)/graphene oxide (GO) nanofibers, which were utilized to fabricate highly sensitive wearable pressure and pyroelectric respiration sensors. This sensor can accurately and rapidly detect pressures as low as 10 Pa with high sensitivity (4.3 V/kPa) (Figure 19a)—a crucial performance metric for wearable sensors. Importantly, the sensor exhibits high sensitivity to bending and stretching motions of the fingers, wrists, and elbows. When attached to the neck, the pressure sensors demonstrate high sensitivity to voice vibrations. Moreover, the device achieves a maximum output power density of approximately 6.2 mW/m^2^ under compressive stress, expanding its range of potential applications. Additionally, doping with GO enhances the pyroelectric energy-harvesting and sensing performance of the device during repeated temperature fluctuations [122].

### 5.3. Sound Sensor

**Figure 20:**
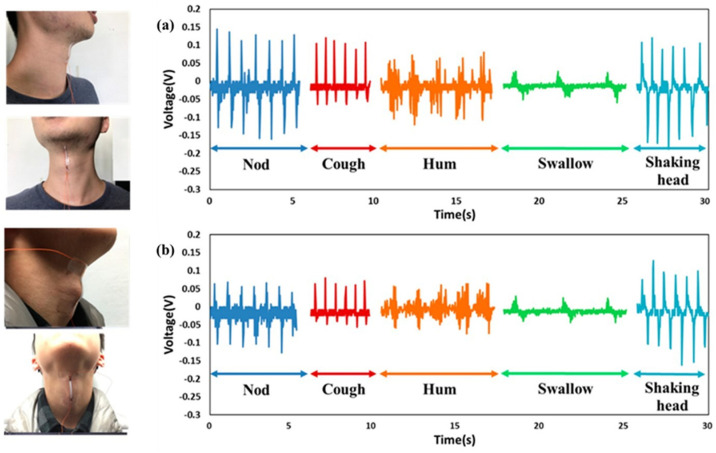
ISSE output voltage according to neck vibration method (nodding, coughing, buzzing, swallowing, head shaking) (**a**) subject, (**b**) other subject; Reproduced with permission from ref. [123]; Copyright 2018, MDPI.

A novel integration of three-dimensional (3D) structures of near-field electrospun PVDF nanomicrofibers (NMFs) was applied to an intelligent self-powered sound-sensing element (ISSE). By employing a 3D architecture with significantly improved piezoelectric output, acoustic energy can be harvested under sound pressure levels exceeding 120 dB SPL, yielding electrical signals of approximately 0.25 V, as shown in Figure 20. Finally, the meticulously crafted ultrathin ISSE, utilizing near-field electrospun piezoelectric fibers, benefits from the capability of direct fabrication onto highly flexible substrates and low cost [113].

An efficient and cost-effective in situ fabrication strategy to construct large-area, high-sensitivity, flexible pressure sensors using electrospun Ce^3+^-doped PVDF/graphene composite nanofibers is presented. The overall device manufacturing process is scalable and allows for large-area integration. The high sensitivity enables the detection of pressures as low as 2 Pa. Moreover, an ultrasensitive pressure sensor based on Ce^3+^-doped PVDF/graphene nanofibers can serve as an effective nanogenerator, generating an output voltage of 11 V with a current density of approximately 6 nA/cm^2^ upon the repeated application of mechanical stress, momentarily lighting up a blue LED. Additionally, for applications involving environmental random vibrations (e.g., wind currents, waterfalls, vehicular transport), the nanogenerator can be integrated with music vibrations to instantly power three blue LEDs, creating an ultrasensitive acoustic nanogenerator (ANG). The outstanding sensing properties, combined with the mechanical flexibility, integrity, and robustness of the nanofibers, enable the real-time monitoring of sound waves and the detection of various types of musical vibrations [124].

### 5.4. Displacement Sensor

A flexible piezoelectric element was developed by manufacturing PVDF (polyvinylidene fluoride) piezoelectric nanofibers using the near-field electrospinning (NFES) method. Innovative screen-printing technology was employed to produce bent electrodes designed with a d33 mode pattern. The electrodes and PVDF nanofibers were affixed to a polyimide film substrate. Compared to piezoelectric ceramics, piezoelectric fibers are more affordable, flexible, and biocompatible. They also exhibit a higher electron density than piezoelectric films, indicating more efficient electromechanical conversion. Accordingly, in this study, a piezoelectric fiber was utilized to fabricate a displacement sensor with bendable electrodes by employing an optimized pattern design to enhance piezoelectric conversion efficiency and sensitivity. The experimental results demonstrate the significance of the electrode type in increasing the output voltage. The new bent electrode induced an average positive voltage of 960.5 mV during the tapping experiment, representing a 59.74% increase in maximum voltage compared to the serial electrode. The positioning accuracy of the displacement sensor is 600 μm, confirming the successful determination of position and validating the displacement detection mechanism [125].

## 6. Conclusions

Advancements in the field of flexible nanogenerators, particularly those composed of PVDF-based polymers fabricated using the electrospinning process, were extensively reviewed. A detailed examination of various studies underscored the significant potential of electrospun PVDF polymers in energy-harvesting applications, attributed to their high piezoelectric performance, flexibility, and durability. Moreover, integrating nanotechnology through electrospinning enhances the surface area and mechanical properties of PVDF fibers, thereby improving their energy conversion efficiency.

The critical role of nanoscale engineering in optimizing the performance of PVDF-based nanogenerators was highlighted. Innovations in fabrication techniques, including tuning electrospinning parameters and incorporating various conductive and piezoelectric materials, have led to notable improvements in output power and device stability. Furthermore, exploring hybrid and composite structures has opened new avenues for developing more efficient, robust, and multifunctional energy-harvesting devices. Finally, the importance of interdisciplinary collaboration in advancing the field of flexible nanogenerators was highlighted, and there are calls for continued research efforts to harness the full potential of electrospun PVDF-based polymers. By leveraging the unique properties of these materials and further refining the electrospinning process, the emergence of more sophisticated and efficient energy-harvesting systems is anticipated in the near future.

Despite progress, challenges such as scalability, long-term stability in real-world conditions, and integration with existing power management systems persist. Future research should focus on addressing these challenges, exploring the potential of new materials and structures, and advancing the integration of flexible nanogenerators into practical applications. Continued innovation in this field holds promise for revolutionizing the harnessing of ambient energy, paving the way for self-powered wearable electronics, smart textiles, and other next-generation technologies.

## Figures and Tables

**Table 1:** Comparison of β-phase contents and crystallinity of random fiber and aligned fiber [82].

$F(\beta )\;\mathrm{and}\;{∆X}_{c}$ Content (%)	Random Fibers			Aligned Fibers		
	Wrinkled	Smooth	Porous	Wrinkled	Smooth	Porous
F(β)	87.53	83.04	79.72	87.57	83.67	81.99
∆Xc	53.27	51.5	49.17	55	52.13	50.1

## Data Availability

The data presented in this study are available upon request from the corresponding author.
